# DNA Hypomethylation Is Associated with Increased Inflammation in Peripheral Blood Neutrophils of Children with Autism Spectrum Disorder: Understanding the Role of Ubiquitous Pollutant Di(2-ethylhexyl) Phthalate

**DOI:** 10.3390/metabo13030458

**Published:** 2023-03-22

**Authors:** Ali A. Alshamrani, Samiyah Alshehri, Sana S. Alqarni, Sheikh F. Ahmad, Hanan Alghibiwi, Naif O. Al-Harbi, Saleh A. Alqarni, Laila Y. Al-Ayadhi, Sabry M. Attia, Ali S. Alfardan, Saleh A. Bakheet, Ahmed Nadeem

**Affiliations:** 1 Department of Pharmacology and Toxicology, College of Pharmacy, King Saud University, Riyadh 11451, Saudi Arabia; 2 Department of Medical Laboratory Science, College of Applied Medical Sciences, King Saud University, Riyadh 11451, Saudi Arabia; 3 Department of Physiology, College of Medicine, King Saud University, Riyadh 11451, Saudi Arabia

**Keywords:** ASD, environmental pollutant, neutrophils, DNMT1, DNA methylation

## Abstract

Autism spectrum disorder (ASD) is a multidimensional disorder in which environmental, immune, and genetic factors act in concert to play a crucial role. ASD is characterized by social interaction/communication impairments and stereotypical behavioral patterns. Epigenetic modifications are known to regulate genetic expression through various mechanisms. One such mechanism is DNA methylation, which is regulated by DNA methyltransferases (DNMTs). DNMT transfers methyl groups onto the fifth carbon atom of the cytosine nucleotide, thus converting it into 5-methylcytosine (5mC) in the promoter region of the DNA. Disruptions in methylation patterns of DNA are usually associated with modulation of genetic expression. Environmental pollutants such as the plasticizer Di(2-ethylhexyl) phthalate (DEHP) have been reported to affect epigenetic mechanisms; however, whether DEHP modulates DNMT1 expression, DNA methylation, and inflammatory mediators in the neutrophils of ASD subjects has not previously been investigated. Hence, this investigation focused on the role of DNMT1 and overall DNA methylation in relation to inflammatory mediators (CCR2, MCP-1) in the neutrophils of children with ASD and typically developing healthy children (TDC). Further, the effect of DEHP on overall DNA methylation, DNMT1, CCR2, and MCP-1 in the neutrophils was explored. Our results show that the neutrophils of ASD subjects have diminished DNMT1 expression, which is associated with hypomethylation of DNA and increased inflammatory mediators such as CCR2 and MCP-1. DEHP further causes downregulation of DNMT1 expression in the neutrophils of ASD subjects, probably through oxidative inflammation, as antioxidant treatment led to reversal of a DEHP-induced reduction in DNMT1. These data highlight the importance of the environmental pollutant DEHP in the modification of epigenetic machinery such as DNA methylation in the neutrophils of ASD subjects.

## 1. Introduction

Autism spectrum disorder (ASD) is a prevalent neuropsychiatric disorder which affects children from a very young age and may persist into adulthood. A subject with ASD shows typical characteristics which include social interaction/communication impairments and stereotypical repetitive/restricted behavioral outcomes [1,2]. A subject with ASD usually experiences a number of difficulties in life—often related to health, employment, and quality of education—all of them ultimately causing huge economic burden on families and the overall healthcare system [3]. The prevalence of ASD has significantly risen in recent times, i.e., from ~2 cases/10,000 (before 1980) to ~15 cases/10,000 (from 1980 to 2010), suggesting an important contribution from environmental and epigenetic factors in this phenomenon [4,5].

Epigenetic modifications such as DNA methylation and histone protein acetylation/deacetylation are known to regulate genetic expression through various mechanisms. DNA methylation may upregulate or downregulate certain genes, depending on the milieu of the immune cell or tissue [6,7,8]. Disruptions in methylation patterns of DNA have been reported to be involved in the pathogenesis of several neurological and immune-mediated mental disorders [8,9,10,11].

DNA methylation is a complex process and involves several steps usually catalyzed by DNA methyltransferases (DNMTs) in association with a cofactor, S-adenosyl-methionine (SAM). DNMT transfers methyl groups donated by SAM onto the fifth carbon atom of the cytosine nucleotide, thus converting it into 5-methylcytosine (5mC), which is usually flanked by a guanine nucleotide (known as CpG sites) in a DNA sequence [7,8,12]. CpG sites are dynamically regulated by different DNMTs, such as DNMT1 and DNMT3s (DNMT3A and 3B), during embryo development, childhood, and adulthood in a cell-dependent manner. Recent studies have shown that changes in DNA methylation caused by DNMTs may be either temporary or permanent depending on the context and the timing. DNMT1 is more active in proliferating cells such as immune cells/keratinocytes, and serves a crucial function [8,13,14]; however, its role in the neutrophils of ASD subjects has not been explored previously.

Phthalates are endocrine-disrupting chemicals that are abundantly present in our surroundings. Phthalates are generally used as plasticizers in a variety of consumer products. One of the most abundant phthalates in food and the environment is di-(2-ethylhexyl)-phthalate (DEHP), which humans may consume through food, drink, and inhalation. Direct skin exposure and the intensive care units in hospitals are important sources as well [15,16,17,18]. DEHP is present in a variety of commercial products, such as beauty and hygiene products, plastic products, food packaging, and medical supplies [17,18]. Several studies in humans and animals have shown that DEHP is capable of modifying epigenetic information through a variety of mechanisms, including DNA methylation modification via DNMTs [19,20,21,22]. However, the role of DEHP in relation to DNMT1 and global DNA methylation in the neutrophils of ASD subjects remains unexplored.

Neutrophils form a crucial part of the innate immune system through multiple mechanisms. They possess oxidative enzymes, such as iNOS, MPO and NADPH oxidase, which kill microorganisms during infection for the healthy maintenance of the immune system. They also trap foreign pathogens through NETosis, which aids in clearance of infection [23,24,25,26]. Neutrophils express inflammatory chemokines/receptors, such as CCR2 and MCP-1, which may help in the amplification of oxidative inflammation during immune-mediated disorders [27,28,29]. All these inflammatory cytokines/receptors existent in neutrophils may be influenced by DNA methylation; therefore, we attempted to explore whether there are differences in DNA methylation patterns and DNMT1 in the neutrophils of ASD subjects as compared with TDC subjects. Further, the effect of the environmental pollutant DEHP on these phenomena in both groups. Our study shows that there is hypomethylation of DNA along with a reduction in DNMT1 expression in the neutrophils of ASD subjects. Hypomethylation is associated with increased CCR2 and MCP-1 expression in the neutrophils of ASD subjects. DEHP affects both these processes in ASD neutrophils, while having negligible effect in TDC neutrophils.

## 2. Materials and Methods

### 2.1. Participants

This investigational study consisted of a total of 52 subjects, of whom 28 were from the ASD group (24 boys and 4 girls; age: 7.55 ± 2.95 years, mean ± SD) and 24 were from the TDC group (20 boys and 4 girls; age: 8.45 ± 3.15 years, mean ± SD). Children with ASD were recruited from the Autism Research and Treatment Center, Faculty of Medicine, King Saud University. The ASD children recruited in this study did not have any history of inflammatory/autoimmune (psoriasis, rheumatoid arthritis), metabolic (phenylketonuria), and neurological/neuropsychiatric (e.g., mental retardation, cerebral palsy, seizures, bipolar disorder, depression) disorders. TDC children were recruited from the Well Baby Clinic, King Khalid University Hospital, Faculty of Medicine, King Saud University, Riyadh, Saudi Arabia, where they usually attended alongside their younger siblings for periodic checkups of their growth indices. TDC children who had any family history of ASD and any of the immunological/neurological disorders listed above were excluded from this study. Both TDC and ASD subjects were healthy and playful at the time of venipuncture and were not taking any vitamin/immunomodulatory supplements. Before the study commenced, the parents/legal guardians of all the children were informed and their written consent was obtained. Approval for the experiments conducted in this study was also obtained from the Research Ethics Committee of the Faculty of Medicine, King Saud University, Riyadh, Saudi Arabia.

### 2.2. Study Measurements

For the diagnosis of ASD, children with ASD in this study were examined for both clinical and neuropsychiatric characteristics by well-trained medical staff guided by a chief physician. Medical staff recorded all the family history along with the physical and neuropsychiatric assessment of the participating children according to the guidelines and recommendations for the diagnosis of autism listed in the 5th edition of the of the *Diagnostic and Statistical Manual of Mental Disorders* (American Psychiatric Association, 2013) [30]. Furthermore, disease severity was categorized by the Childhood Autism Rating Scale (CARS), which assesses a child on a scale from 1 to 4 in 15 different areas, as detailed previously in the literature [31]. Based on the CARS scoring, the ASD group was further divided into two subgroups: the mild–moderate group (*n* = 16; M-ASD group; CARS score: 30–36) and the severe group (*n* = 12; S-ASD group; CARS score: 37–60).

### 2.3. Separation of Peripheral Blood Neutrophils

Peripheral blood neutrophils were separated using density gradient centrifugation as detailed in previous studies [25,32]. Briefly, blood was collected through venipuncture in an acid-citrate-dextrose Vacutainer tube (BD Biosciences; Franlin Lakes, NJ, USA). This was followed by separation of polymorphonuclear neutrophils (PMNs) from the whole blood using Ficoll-Paque solution (1.077 g/mL; Sigma-Aldrich, St. Louis, MO, USA) as part of the density gradient centrifugation process. This process separates blood leukocytes into different bands, with PMNs staying at the bottom layer of the tube. Next, the PMN layer was processed using the dextran sedimentation method to isolate neutrophils with greater than 95% purity, which were subsequently utilized for different molecular analyses.

### 2.4. Assessment of Overall DNA Methylation

Global DNA methylation in neutrophils isolated from ASD and TDC subjects was measured using the MethylFlash™ Global DNA Methylation Kit (EpiGentek, Farmingdale, NY, USA). This kit measures an overall % of 5-mC in DNA, which is an indicator of DNMT activity. Data are presented as % of 5-mC DNA relative to the total DNA.

### 2.5. Flow Cytometry

Flow cytometry was conducted on peripheral blood collected from the TDC and ASD groups as described in a previous study [25]. Blood leukocytes containing neutrophils (after RBC lysis) were first immunostained with anti-CD15 cell-surface antibody (marker of neutrophils) linked to FITC/PE/APC (Biolegend, San Diego, CA, USA). After conducting the usual steps of permeabilization and fixation, the neutrophils were again immunostained with specific monoclonal/polyclonal antibodies against intracellular proteins of interest, such as DNMT1, CCR2, MCP-1, and nitrotyrosine linked to APC/FITC/PE (Biolegend, San Diego, CA, USA; Santa Cruz Biotech, Dallas, TX, USA). Each sample containing blood leukocytes labeled with anti-CD15 and other antibodies was run in a flow cytometer (Cytomics FC500 software, Beckman Coulter, Indianapolis, IN, USA), and 10,000 events were acquired for analysis of cell-surface and intracellular proteins as described in [25].

### 2.6. Neutrophils Cell Culture

Freshly isolated neutrophils from the TDC and ASD groups were placed in 12-well culture plates with or without DEHP (10 uM final concentration) and antioxidant, N-acetyl cysteine (1 mM final concentration) in RPMI-1640 medium (Invitrogen, Waltham, MA, USA) provided with heat-inactivated FBS (Gibco, Grand Island, NY, USA), for 12 h. This was followed by different biochemical/molecular analyses according to the set protocols listed above.

### 2.7. Statistical Analysis

The data were presented as mean ± SEM. All molecular and biochemical parameters were analyzed using one-way ANOVA (analysis of variance) followed by Tukey’s post hoc tests. The level of statistical significance was set at *p* < 0.05 for differences between the groups. All the statistical analyses were performed using the Graph Pad Prism 9 statistical package (GraphPad Software, San Diego, CA, USA).

## 3. Results

### 3.1. Hypomethylation of DNA in Neutrophils of Children with ASD

**Figure 1:**
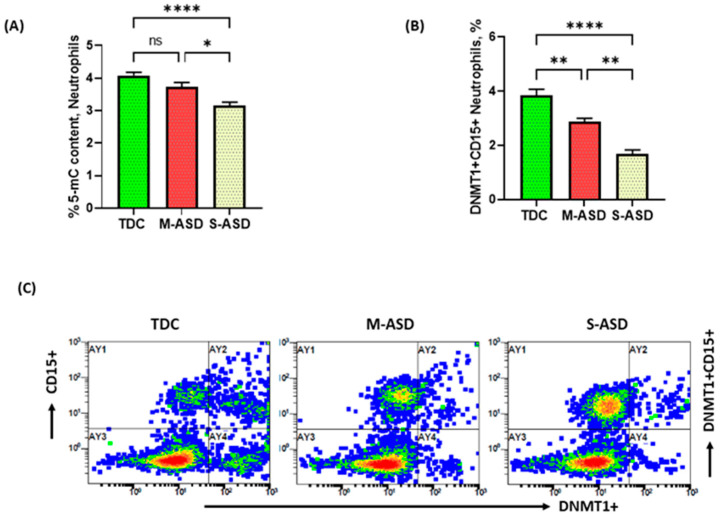
Methylation of DNA and DNMT1 expression in neutrophils of ASD and TDC subjects. (**A**) Global DNA methylation levels in neutrophils, (**B**) DNMT1 expression in neutrophils, and (**C**) illustrative flow diagram displaying DNMT1+CD15+ neutrophils. Values are presented as mean ± SEM, *n* = 24–28/group. * *p* < 0.05; ** *p* < 0.01; **** *p* < 0.0001; ns = not significant.

It is well known that epigenetic modulation may affect the inflammatory potential of immune cells; therefore, we explored the levels of global methylation and DNMT1 expression in major systemic innate cells, i.e., the neutrophils of ASD and TDC subjects. Our data show that the neutrophils of ASD subjects have hypomethylated DNA levels neutrophils, as reflected by the global methylation pattern of the DNA (Figure 1A). DNMT1 expression, the main enzyme responsible for methylation of DNA, was also measured. Our data show decreased DNMT1 expression in the CD15+ neutrophils in ASD subjects as compared with the TDC group (Figure 1B,C). In addition, ASD subjects were sectioned into M-ASD and S-ASD subjects. Our data additionally exhibit that the S-ASD subjects have a further decrease in the global methylation DNA pattern in the neutrophils and in % of DNMT1+ in the CD15+ neutrophils as compared with the M-ASD group (Figure 1A). These data show that the DNA in the neutrophils is in a hypomethylated state in the ASD subjects, which could be due to reduced DNMT1 expression.

### 3.2. Increase in Inflammatory Mediators in Neutrophils of Children with ASD

**Figure 2:**
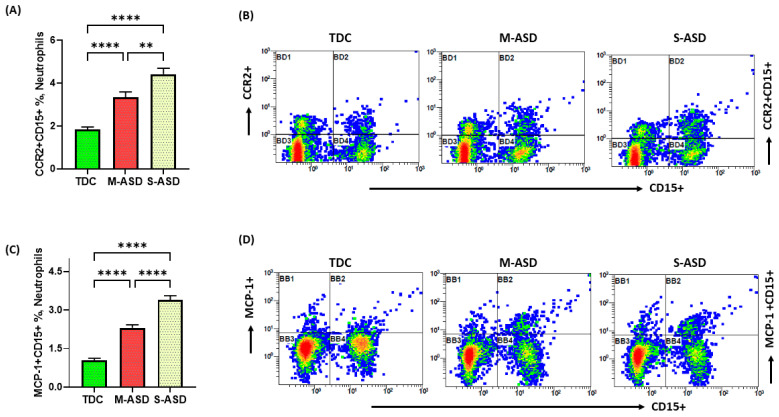
CCR2 and MCP expression in neutrophils of ASD and TDC subjects. (**A**) CCR2 expression in neutrophils, (**B**) illustrative flow diagram displaying CCR2+CD15+ neutrophils, (**C**) MCP-1+ expression in neutrophils, and (**D**) illustrative flow diagram displaying MCP-1+CD15+ neutrophils. Values are presented as mean ± SEM, *n* = 24–28/group. ** *p* < 0.01; **** *p* < 0.0001.

Next, we wanted to examine whether DNA hypomethylation in the neutrophils was associated with inflammatory mediators in the ASD group. For this purpose, MCP-1 and CCR2 were measured in the neutrophils of the ASD and TDC groups. Our data exhibit that the ASD subjects have increased inflammatory markers in the neutrophils as mirrored by an increased % of MCP-1+CD15+ and CCR2+CD15+ neutrophils when compared with the TDC group (Figure 2A–D). Our data further exhibit that the S-ASD subjects have a further increase in % of MCP-1+CD15+ and CCR2+CD15+ neutrophils as compared with the M-ASD group (Figure 2). These data suggest that inflammatory mediators are upregulated in the neutrophils of ASD subjects and that their expression increases with increasing disease severity.

### 3.3. DEHP Downregulates DNMT1 Expression in Neutrophils of Children with ASD

**Figure 3:**
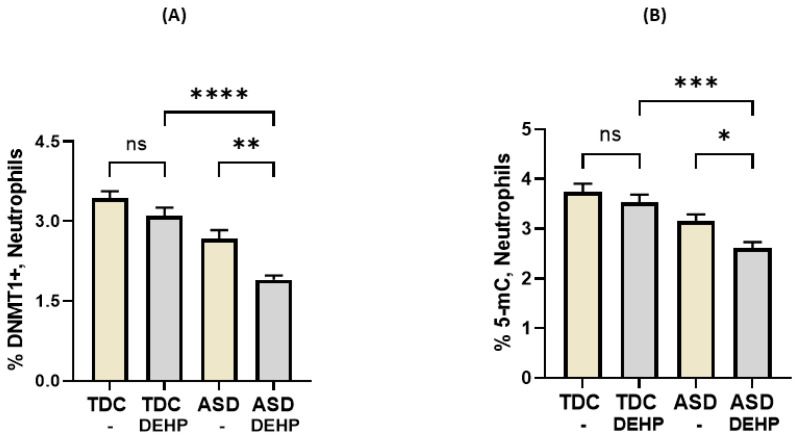
Effect of DEHP on DNMT1 expression and DNA methylation in neutrophils of ASD and TDC subjects. (**A**) Percentage of DNMT1 expression in neutrophils and (**B**) global DNA methylation levels in neutrophils. Values are presented as mean ± SEM, *n* = 15/group. * *p* < 0.05; ** *p* < 0.01; *** *p* < 0.001; **** *p* < 0.0001; ns = not significant.

DEHP is known to modulate cellular responses through epigenetic mechanisms; therefore, we explored its role in relation to DNMT1 expression and global DNA methylation in the neutrophils of ASD and TDC children. Our results reveal that treatment of the neutrophils with DEHP causes a reduction in the expression of DNMT1 in the ASD neutrophils, but not in the TDC neutrophils, as reflected by a decreased % of DNMT1+ neutrophils (Figure 3A). Consequently, global DNA methylation was also lower in the ASD neutrophils after treatment with DEHP, as depicted by a decreased % of 5-mC levels (Figure 3B). Global DNA methylation levels were similar before and after treatment with DEHP in the TDC neutrophils. These data imply a role for DEHP in the reduction of DNA methylation in the ASD neutrophils.

### 3.4. DEHP Upregulates Inflammatory Mediators in Neutrophils of Children with ASD

**Figure 4:**
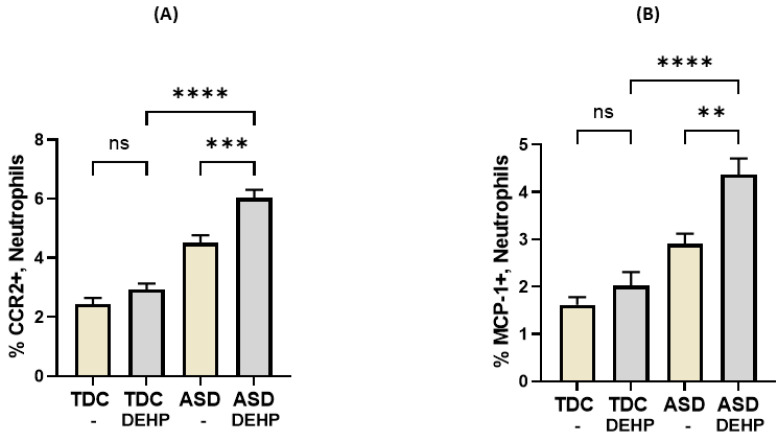
Effect of DEHP on CCR2 and MCP-1 expression in neutrophils of ASD and TDC subjects. (**A**) Percentage of CCR2 expression in neutrophils and (**B**) percentage of MCP-1 expression in neutrophils. Values are presented as mean ± SEM, *n* = 15/group. ** *p* < 0.01; *** *p* < 0.001; **** *p* < 0.0001; ns = not significant.

Next, the effect of DEHP on CCR2 and MCP-1 in the neutrophils of ASD and TDC children was examined. Our results show that treatment of neutrophils with DEHP causes upregulation in expression in the CCR2 and MCP-1, but not in the TDC neutrophils, as mirrored by an increased % of CCR2+ and % of MCP-1+ neutrophils (Figure 4A,B). Levels of inflammatory markers were similar before and after treatment with DEHP in the TDC neutrophils. Further, the percentage of nitrotyrosine expression also increased significantly in the neutrophils of ASD subjects after DEHP treatment, while having insignificant effect in the TDC neutrophils (data not shown). These data imply a role for DEHP in the elevation of inflammatory signaling in ASD neutrophils.

### 3.5. Antioxidant Treatment Reverses DEHP-Induced Changes in DNMT1 Expression and MCP-1 in Neutrophils of Children with ASD

**Figure 5:**
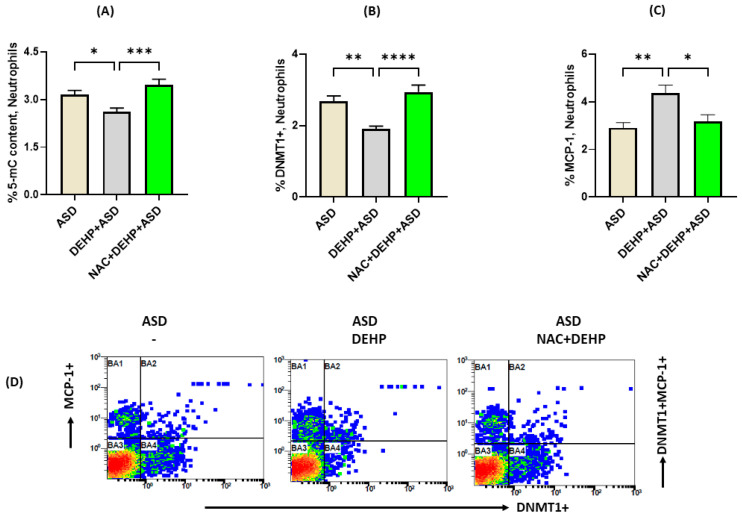
Effect of antioxidant, N-acetyl cysteine, on DEHP-mediated modulation of DNMT1/DNA methylation and MCP-1 expression in ASD neutrophils. (**A**) Global DNA methylation levels in neutrophils, (**B**) percentage of DNMT1 expression in neutrophils, (**C**) percentage of MCP-1 expression in neutrophils, and (**D**) illustrative flow diagram displaying double immunostaining of DNMT1+ and MCP-1+ neutrophils. Values are presented as mean ± SEM, *n* = 15/group. * *p* < 0.05; ** *p* < 0.01; *** *p* < 0.001; **** *p* < 0.0001.

Finally, the cause for DEHP-induced reduction in DNMT1 expression was explored. For this purpose, we first investigated whether antioxidant treatment would be able to reverse DEHP-induced effects on DNA methylation, as oxidant radicals produced in ASD neutrophils may be responsible for modulation of DNMT1. Our data revealed that DEHP-mediated reductions in % 5-mC levels in ASD neutrophils were inhibited by pretreatment with the antioxidant NAC (Figure 5A). Furthermore, the decrease in MCP-1 expression in ASD neutrophils by NAC pretreatment was paralleled by a marked increase in DNMT-1 expression, as shown by a significant decrease in % of MCP-1+ neutrophils and an increase in % of DNMT+ neutrophils, respectively (Figure 5B,C). Overall, our results show that DNA methylation and inflammatory markers are modified by the environmental pollutant DEHP in the neutrophils of children with ASD.

## 4. Discussion

ASD is a multifactorial disorder in the pathogenesis of which environmental, immune, and genetic factors act in concert to play a crucial role. The innate immune system is the first line of defense against invading pathogens; however, recently it has been implicated in the pathogenesis of several immune-mediated disorders including ASD. An important component of the innate immune system are neutrophils, which are known for their varied functions that include the killing of pathogens through oxidative burst and the release of proteases and inflammatory mediators [5,23]. These mediators are important when there is an infection but can be detrimental if there is excessive activation of the neutrophilic function in the absence of infection, as it can cause inflammation [23,25,33]. DNA methylation patterns play a crucial role in the maintenance of a healthy immune system. However, DNMT1 signaling in neutrophils has remained unexplored in relation to ASD. Moreover, how the ubiquitous plasticizer DEHP modulates DNA methylation in the neutrophils of ASD subjects also remains uninvestigated. Our data show that DNMT1, the main enzyme responsible for the maintenance of DNA methylation patterns is decreased in the ASD neutrophils as compared with the TDC neutrophils. This was reflected by global hypomethylated DNA levels in the ASD neutrophils.

Most common epigenetic modifications involve DNA methylation and histone acetylation/deacetylation. DNMTs are known enzymatic “writers” that add a methyl group to the fifth position in the cytosine moiety in the presence of the methyl donor SAM, thus establishing specific methylation patterns during cell division which may be temporary or permanent in nature [34]. The methylation patterns in CpG islands are known to occur more commonly in promoter regions of the DNA and control transcription through controlling accessibility of transcription factors when methylated [35]. DNA methylation patterns may be impacted by several factors, such as diet, radiation, and inflammation, ultimately causing activation or suppression of certain genes that play an important role in disease development or pathogenesis [8,22,36]. DNMT-1 plays a quintessential role in maintaining DNA methylation patterns under healthy and pathologic conditions [35,37]. Our study shows downregulation of DNMT1 expression in the ASD neutrophils.

DNA hypomethylation has been reported in different immune-mediated disorders, including ASD [12,13,37,38,39]. A past study showed hypomethylated CG sites in neutrophils of lupus patients as compared with controls, indicating hypomethylation of DNA with disease pathogenesis [40]. Global hypomethylation of blood cells specifically in monocytic cells was independently associated with the risk of CAD in the Chinese Han population [6]. Our study also exhibited global DNA hypomethylation as mirrored by % of 5-mC in neutrophils of ASD subjects. Global DNA hypomethylation in brain and systemic circulation of ASD patients has also been shown previously [34,41,42,43]. Our study showed hypomethylated DNA along with increased expression of inflammatory genes in the neutrophils of ASD subjects.

Modification in DNA methylation motifs has the potential to change the gene expression, and hence cell function, through transcriptional machinery regulation. It is reported that DNA methylation is commonly linked to gene silencing, while hypomethylation can lead to gene expression [8,10]. Transcriptional activation of certain genes in the neutrophils may be preceded by hypomethylation of their promoters. DNA hypomethylation has been reported to be a permissive signal for gene expression. In different immune cells such as T cells and neutrophils, cell-type-specific hypomethylated regions have been shown to be linked to higher gene transcription levels, which play a crucial role in maintaining inflammatory signals of the immune system [26]. Another recent report has shown that DNMT1 deficiency and DNA hypomethylation in vivo cause autoinflammation of the skin through upregulation of genes in keratinocytes which are involved in inflammation and chemotaxis [13]. DNA hypomethylation at CpG sites in leukocytes of whole blood at birth was correlated with increased inflammatory indices and childhood asthma [44]. Similarly, hypomethylated DNA in the neutrophils in this study was associated with overexpression of chemoattraction-related markers such as CCR2 and MCP-1, which participate in amplification of inflammatory immune reactions.

Usage of DEHP as a plasticizer reaches several million tons/year, and it is utilized in numerous household/industrial products ranging from floor materials and plastic toys to cosmetic products and medical supplies [16,45]. Therefore, exposure of humans throughout the world to DEHP is quite prevalent through water, air, and food, which may impact the human immune system [16,45]. Increased DEHP exposure has been reported in several studies which showed the presence of DEHP metabolites in different body fluids in ASD subjects [46,47,48]. Increased DEHP exposure may cause modification in the immune responses in ASD subjects, which could be due to epigenetic mechanisms. However, this has not previously been explored with respect to DNA methylation in the neutrophils of ASD subjects.

DEHP may influence DNA methylation in neutrophils through modulation of oxidative inflammation created by disturbance in overall redox homeostasis. Phagocytic cells such as neutrophils produce large amounts of oxidants, such as superoxide radical, hydrogen peroxide, and peroxynitrite, which may further give rise to other more potent oxidants through MPO-mediated reactions [25,27,49,50]. These oxidants include hypochlorous acid, which may give rise to secondary oxidants such as chloramines. These oxidants can selectively target several redox groups, such as thiols and methionine [51,52]. Methionine is required for synthesis of SAM, thereby serving a central role in metabolic pathways leading to DNA methylation. It has been reported in the past that DNMT1 is susceptible to inactivation by oxidative damage in immune and non-immune cells. Furthermore, methionine oxidation by neutrophil oxidants may also limit SAM synthesis, thereby compromising the enzymatic activity of DNMT1 [14,51,52]. Therefore, the oxidative milieu created by DEHP within the ASD neutrophils may inactivate DNMT1 and subsequent DNA hypomethylation, which ultimately may be responsible for overall amplification of inflammation in the ASD neutrophils.

Our findings imply that DNA hypomethylation by environmental pollutants such as DEHP may perturb the epigenetic programming of neutrophils, which may enhance systemic/neurological inflammation in ASD subjects. Since CCR2/MCP-1 is involved in mediating transmigration of neutrophils to different tissues, it is likely that DEHP plays a significant role in causing neurological changes in ASD [28,29,53,54]. MCP-1 levels have previously been shown to be elevated in the systemic compartments of ASD subjects [55]. Further, a blockage of the CCR2/MCP-1 chemoattraction pathway was recently shown to cause a reduction in ASD-type phenotypes in animals [56].

Other environmental pollutants reported to be increased in children with ASD, such as lead and mercury, also need to be investigated with respect to DNA methylation in the neutrophils [7,22,57]. Moreover, DNA methylation patterns in other immune cells, such as T cells, B cells, and monocytes, also need to be explored, as they will provide crucial information about the impact of different environmental pollutants on epigenetic programing. This information will be critical for counteracting pollution-induced dysfunction in the immune systems of children with ASD.

The cause-and-effect relationship between environmental pollutants such as DEHP and DNA methylation modulation in the neutrophils of children with ASD is an area of considerable interest. However, this is difficult due to the nature of the pollutants that are present in the human environment from the time of birth, which is why most studies report correlation statistics. In our opinion, animal studies performed in a controlled environment and controlled genetic background are better for ascertaining such a cause-and-effect relationship.

In summary, our results exhibit that there is hypomethylated DNA in ASD neutrophils, which is associated with elevated CCR2/MCP-1 expression. Exposure of ASD neutrophils to DEHP results in a further increase in CCR2/MCP-1 expression, probably through downregulation of DNMT1 in ASD subjects. Global DNA hypomethylation in the neutrophils caused by the environmental pollutant DEHP may lead to elevated inflammation, which may contribute to the progression of ASD.

## Data Availability

The data presented in this study are available in article. The authors confirm that all data underlying the findings are fully available without restriction. All relevant data are within the paper.
